# Ductal carcinoma *in situ* of the breast: morphological and molecular features implicated in progression

**DOI:** 10.1042/BSR20130077

**Published:** 2014-01-17

**Authors:** Dirce M. Carraro, Eliana V. Elias, Victor P. Andrade

**Affiliations:** *Laboratory of Genomics and Molecular Biology, International Center of Research, A.C. Camargo Cancer Center, São Paulo, SP 01509-900, Brazil; †National Institute of Science and Technology in Oncogenomics (INCITO), São Paulo, SP 01509-900, Brazil; ‡Department of Anatomical Pathology, A.C. Camargo Cancer Center, São Paulo, S.P. 01509-900, Brazil

**Keywords:** Breast cancer, cancer progression, Ductal carcinoma *in situ* (DCIS), epithelial cells, invasive breast carcinoma (IBC), microenvironment, BM, basal membrane, CAFs, carcinoma-associated fibroblasts, CC, comedocarcinoma, DCIS, ductal carcinoma *in situ*, DCIS-IBC, ductal carcinoma *in situ* with co-existing invasive breast carcinoma, ER, estrogen receptor, HG, high grade, IBC, invasive breast carcinoma, LCM: laser capture microdissection, LG, low grade, MEC, myoepithelial cell, miRNA, microRNAs, MMP, matrix metalloproteinase, mTOR, mammalian target of rapamycin, PR, progesterone receptor

## Abstract

The spread of mammographic screening programmes around the world, including in developing countries, has substantially contributed to the diagnosis of small non-palpable lesions, which has increased the detection rate of DCIS (ductal carcinoma *in situ*). DCIS is heterogeneous in several ways, such as its clinical presentation, morphology and genomic profile. Excellent outcomes have been reported; however, many questions remain unanswered. For example, which patients groups are overtreated and could instead benefit from minimal intervention and which patient groups require a more traditional multidisciplinary approach. The development of a comprehensive integrated analysis that includes the radiological, morphological and genetic aspects of DCIS is necessary to answer these questions. This review focuses on discussing the significant findings about the morphological and molecular features of DCIS and its progression that have helped to uncover the biological and genetic heterogeneity of this disease. The knowledge gained in recent years might allow the development of tailored clinical management for women with DCIS in the future.

## CONCEPT AND EPIDEMIOLOGY

DCIS (ductal carcinoma *in situ*), also referred to as non-invasive or intra-ductal cancer, is defined as a neoplastic proliferation of epithelial cells confined to the ductal–lbular system and is characterized by subtle to marked cytological atypia as well as an inherent (but not necessarily obligate) tendency to progress to IBC (invasive breast cancer) [1].

DCIS is typically non-palpable, asymptomatic and discovered incidentally as suspicious (pleomorphic, grouped, linear or segmental) microcalcifications on routine mammographic screening or adjacent to other lesions in the breast [2]. DCIS had been considered rare (2–3%) in the pre-mammographic era [3] but now represents a high proportion (20–25%) of newly diagnosed breast cancers with age-incidence rates ranging from 0.6 to 1.3 per 1000 screening examinations in women aged 40–49 and 70–84 years, respectively [4].

Older age, benign breast disease, a family history of breast cancer in first-degree relatives, reproductive factors (such as nulliparity and older age at the time of the first full-term pregnancy), late age of menopause and long-term use of postmenopausal hormone-replacement therapy are risk factors associated with an increased incidence of DCIS. Genetic factors, such as germline mutations in one of two breast cancer susceptibility genes, *BRCA1* and *BRCA2*, increase the likelihood of developing DCIS [5]. Demographic data predict that 5% of women with DCIS carry a germline mutation in both genes [6].

## CLINICAL RELEVANCE

**Figure 1:**
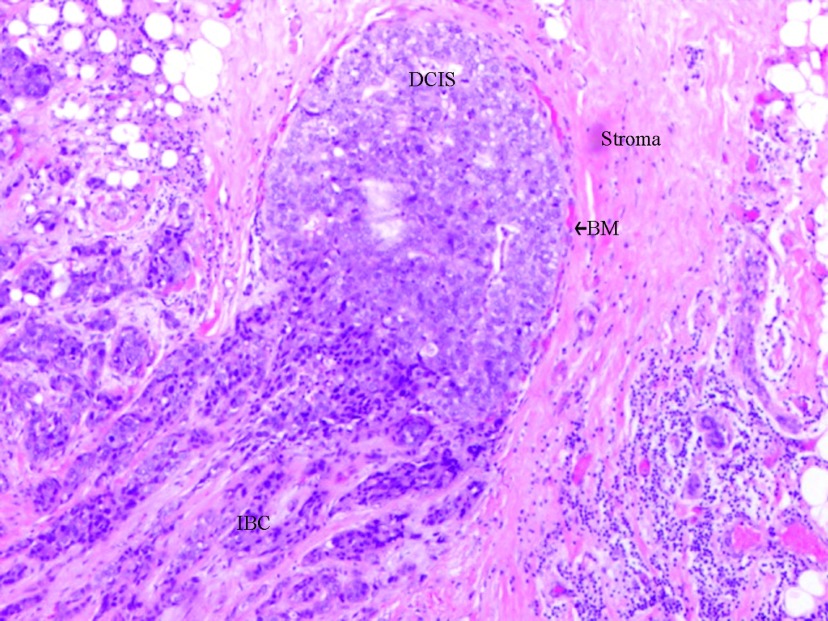
DCIS with invasion In this picture is possible to see the invasive component originated directly from the DCIS (Haematoxylin, original magnification ×200). DCIS, ductal carcinoma *in situ*; IBC, invasive breast cancer; BM, basal membrane.

Although DCIS is non-lethal, the evidence that DCIS is a true precursor of IBC is indirect but convincing: IBC is rarely seen without adjacent DCIS [7]; women diagnosed with DCIS carry a 10 times higher risk of developing ipsilateral invasive breast cancer if left untreated [8]; DCIS and IBC from the same patient share similar genetic features [9] and molecular abnormalities [10–16]; animal models progress from *in situ* to invasive disease [17]; microscopic examinations show the ruptured BM (basal membrane) allowing the invasive cancer to merge with an intra-ductal component (Figure 1); and the risk factors for both DCIS and IBC are similar [18].

The long-term natural history of DCIS is poorly understood and debated in the current literature. The potential for progression to invasive carcinoma varies among the histological types of DCIS, and the current understanding of the biology and clinical behaviour of these lesions remains incomplete, making it difficult to understand the real relationship between DCIS and IBC [19]. Between 14 and 50% of DCIS lesions are estimated to progress to invasive lesions if left untreated [20]. To date, neither the histopathological classification nor the conventional biomarkers can accurately predict whether DCIS lesions can invade the surrounding tissue and consequently progress to metastatic disease [21]. Many efforts have been made to properly classify the risk of progression of DCIS using morphological and molecular features.

## MORPHOLOGICAL CLASSIFICATION AND IMMUNOPHENOTYPE

DCIS shows multifaceted morphologies and varies significantly with respect to nuclear atypia and architectural pattern. These morphological patterns have some clinical implications, such as risk of recurrence, time to recurrence after surgical resection and time to progression to invasive disease. Moreover, the treatment options vary across the subtypes of DCIS. Many classification systems have been proposed, but none has yielded a standardized method to categorize DCIS [22].

Most systems recognize at least three types of DCIS while ignoring small differences: LG (low grade)-DCIS, HG (high-grade) non-comedo and HG comedo-carcinoma (grouped as HG-DCIS). The intermediate grade of DCIS can be distinguished microscopically; it is a separate category in some but not all grading systems and is frequently found in association with either LG-DCIS or HG-DCIS. LG-DCIS and HG-DCIS are rarely found together, and LG-DCIS rarely progresses to HG-DCIS.

LG-DCIS is characterized by monotonous cell proliferation; nuclear size approximates that of a normal ductal cell with a variety of architectural patterns, including cribriform, micropapillary, solid and papillary growth. Cells are well-polarized, and mitotic figures are rare. Punctate necrosis may be found, but large foci of necrosis are uncommon and should not be more than focal in LG-DCIS. Small laminated microcalcifications are a common finding.

HG-DCIS shows large and pleomorphic, hyperchromatic nuclei that sometimes exhibit prominent nucleoli. The nuclear size is variable, and mitotic cells may be numerous. Cells may show a loss of polarity, and nuclei show up to 3-fold variations in size. Necrosis with cellular debris is a common finding in small and large foci. Architecturally, HG-DCIS is variable but is more frequently solid, although a single layer of highly atypical cells is sufficient to diagnose HG-DCIS. Amorphous calcifications associated with necrotic foci are common.

Comedocarcinoma *in situ* (CC-DCIS) is a special type of HG-DCIS. CC-DCIS is diagnosed from particular findings at the clinical, gross and microscopic levels with clinical implications. CC-DCIS is the only DCIS that is grossly observable; therefore, it was over-represented in the breast cancers identified in the pre-mammographic era. The term comedo refers to a yellowish creamy necrotic material oozed from the ducts that resemble comedones. Histologically, this subtype is characterized by enlarged ducts filled with necrotic debris surrounded by zero to a few layers of highly atypical epithelial cells and numerous mitotic figures. Necrosis usually spans more than 90% of the duct cross section. The abundant necrotic material calcifies and appears as large linear branching calcifications on the mammogram.

Currently, when DCIS is diagnosed by core needle biopsy, it should be followed by surgical resection because of a 30% risk of underestimating an invasive carcinoma adjacent to the biopsied area. When DCIS is diagnosed on surgical specimens, some variables are important to the clinical decision-making process and should be cited by the pathologist. These variables are as follows: DCIS size, status of surgical margins and the distance to DCIS (as a specific size as opposed to vague descriptions such as ‘close to’, ‘approaching’, and ‘almost touching’), the presence of one or more foci, type and extension of necrosis, DCIS grade and hormone receptor status [1].

Commonly used markers in DCIS include the ER (oestrogen receptor) and PR (progesterone receptor). Less commonly used markers include the epidermal growth factor receptor family member 2 [ERBB2, HER2 (human epidermal growth factor receptor) or HER2/neu], the androgen receptor and TP53. Hormone receptors are expressed in approximately 40% of patients with DCIS [23]. Anti-oestrogen therapies, such as tamoxifen, inhibit the mitogenic activity of DCIS cells and have also been observed to contribute to reducing the risk of recurrence in patients with ER-positive DCIS [24].

ERBB2 is a transmembrane protein with a tyrosine kinase cytoplasmic domain. It is involved in proliferative and anti-apoptotic triggering signals as well as activation pathways such as MAPK (mitogen-activated protein kinase), PI3K (phosphoinositide 3-kinase)/Akt (protein kinase B) and mTOR (mammalian target of rapamycin). Thus, ERBB2 plays an important role in tumour development and progression [25]. Studies have shown that 50–60% of ERBB2/HER2 amplification/overexpression in DCIS is associated with poorly differentiated lesions and the high-grade comedo subtype [26]. However, ERBB2/HER2 expression/amplification status is not currently considered in the decision-making process for DCIS.

## DCIS TREATMENT

The most important goal in treating DCIS is to prevent tumour recurrence and the development of invasive disease. To reduce morbidity and achieve high cure rates, most DCIS patients have been treated by a combination of surgery and postoperative radiation followed by endocrine therapy if the ER is detected by immunohistochemistry.

A few decades ago, the treatment for DCIS was mastectomy with axillary dissection. Although this approach resulted in a cure rate exceeding 99%, the morbidity and aesthetic aspects forced surgeons to use more conservative options. However, patients that exclusively underwent this treatment modality can experience disease recurrence. Several clinical trials have compared surgery with radiotherapy to surgery without radiotherapy. All have concluded that radiotherapy reduces the rates of recurrences by 50% in patients undergoing breast-conserving therapy [29]. However, these trials did not attempt to discriminate patients who did not recur who would otherwise be candidates for avoiding radiation. A large prospective, multi-institutional trial conducted in Europe demonstrated very low rates of 5-year local recurrence when the LG-DCIS was completely excised (margins >3 mm) even without radiotherapy [30]. Given this complexity, different treatment approaches for different types of LG-DCIS may be indicated to avoid overtreatment in many women with LG-DCIS.

ER expression is a predictive marker of the effectiveness of tamoxifen in the treatment of DCIS [31]. However, as expected, ER-negative DCIS did not benefit from that approach and consequently a continuous effort have been done for properly treating high-risk patients with ER-negative. Recently, it was proposed that trastuzumab, a monoclonal antibody against human epidermal growth factor receptor (HER)-2/neu, could be effective in the treatment of high-risk ER-negative, HER2-positive DCIS patients, in preventing the transition of DCIS to IBC [32]. The GeparQuattro study showed that HER2 overexpressing cases of IBC plus DCIS were less responsive to chemotherapy and trastuzumab than pure IBC cases. However, in about 50% of the cases, DCIS adjacent to IBC completely disappeared after neoadjuvant treatment with trastuzumab [33]. Kuerer et al., did not find any significant clinical, histological or apoptotic changes using a single-dose monotherapy with trastuzumab for patients with HER2-positive DCIS. However, this treatment was able to include T-cell-dependent humoral immunity [34]. Considering the fact that some patients develop resistance to trastuzumab-based therapy, possibly because of the crosstalk between receptor and amplified HER2 signalling, trastuzumab in combination with the HER2 inhibitor Lapatinib have also been proposed [35]. In addition, several other strategies to overcome resistance are in different phases of development such as treatment with pertuzumab, T-DM1 and mTOR inhibitors [36]. However, it is still not proved whether ER-negative, HER2-positive DCIS patients would really benefit from HER2-inhibitor treatments. HER2 testing has been increasingly performed in women with DCIS but the recent 2013 ASCO/CAP guidelines for HER2 evaluation on breast cancer do not recommend that it should be tested routinely or that overexpressing DCIS should be treated with trastuzumab [37].

## MOLECULAR AND GENETIC FEATURES OF DCIS AND ITS PROGRESSION

Over the past three decades, the preliminary delineation of the biology and pathology of cancer has become possible. The carcinogenic process is widely hypothesized to consist of multiple steps in which a set of events contributes to cell transformation and subsequent malignant stages. During tumour progression, the primary tumour cells may lose the ability to adhere and initiate the process of invasion through the basement membrane in their tissue of origin. This invasion includes leakage to the bloodstream or lymphatic system and the formation of proliferative areas in other tissues to conclude the metastatic process [38].

Despite the fact that DCIS is considered a non-invasive cancer with favourable prognosis, numerous studies of human and mouse DCIS lesions have shown that DCIS lesions contain carcinoma precursor cells and that the malignant phenotype is predetermined at the premalignant stage [39].

In recent years, scientists have taken advantage of sensitive technologies for the assessment of molecular and genetic modifications at the cellular level to uncover numerous biological features involved in DCIS progression. The use of laser microdissection [LCM (laser capture microdissection)] to capture defined cell populations from a complex solid tissue has been crucial in the assessment of molecular alterations between ductal epithelial cells and cells that have extravasated from the mammary duct. Using LCM in combination with gene expression analysis, several groups investigated the early molecular alterations in epithelial cells that may trigger the progression of DCIS to IBC [10,11,13,40].

Epithelial cells from DCIS and epithelial cells from an invasive component that co-exists in the same lesion [DCIS-IBC (ductal carcinoma *in situ* with co-existing invasive breast carcinoma)] have shown negligible molecular differences, suggesting that molecular abnormalities for the development of an invasive phenotype are already present in pre-invasive epithelial cells [10,11,13,14,40]. The majority of gene expression differences were observed between cells captured from both intraductal components–pure DCIS and the *in situ* component of DCIS-IBC. This finding further reinforces that the molecular alterations are already present before the lesion exhibits morphological changes [10]. Interestingly, a remarkable down-regulation of genes seems to occur in the epithelial cells of both intraductal lesions, from pure DCIS to the *in situ* component of DCIS-IBC [10], suggesting that the DNA hypermethylation of gene promoters may play a role in this step of DCIS progression. This predominant down-regulation has not been observed between cells from DCIS and cells from IBC [13,41–44]. Abnormal methylation, such as DNA hyper-methylation of tumour suppressor genes, is a powerful molecular mechanism by which cancer can be triggered [45,46] and might be associated to pure DCIS progression.

**Figure 2:**
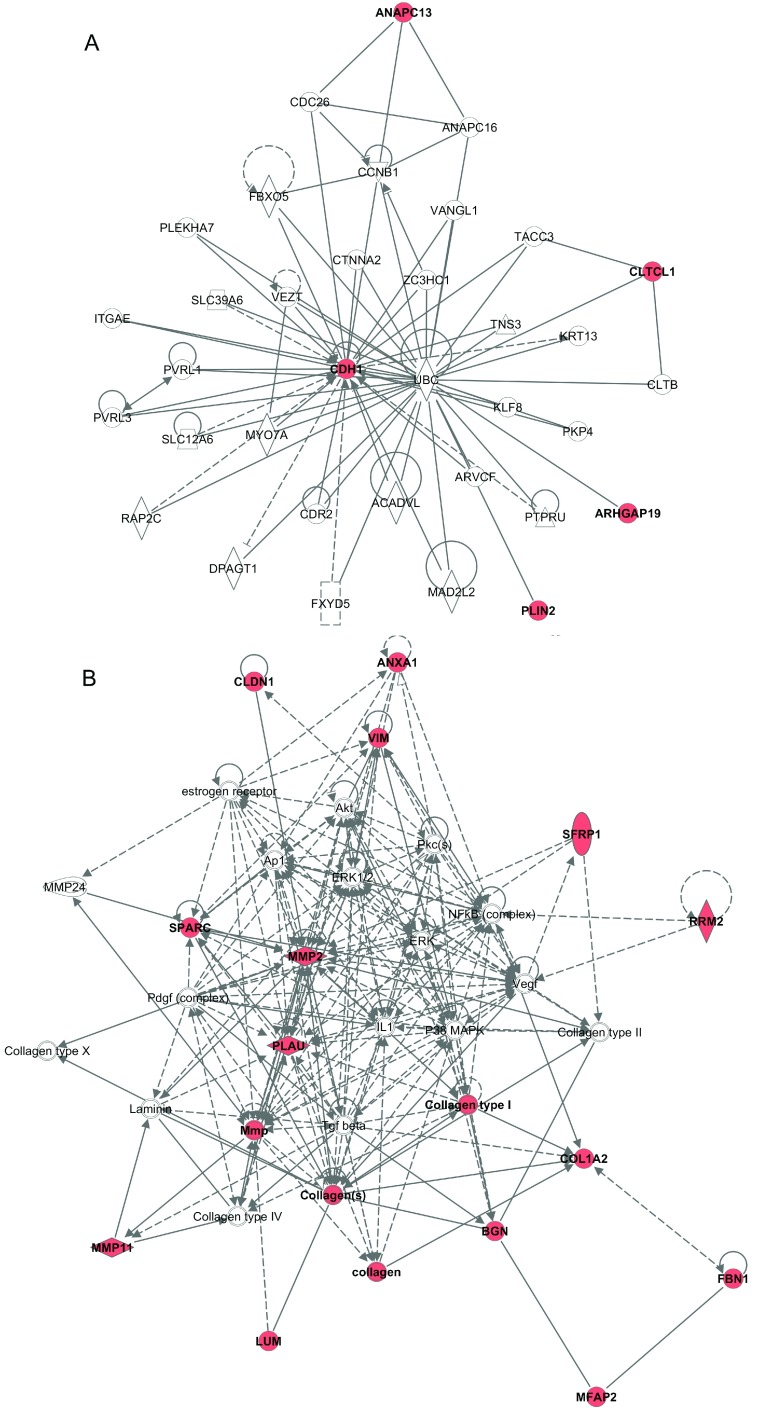
IPA network diagram illustrating annotated interactions between genes that were up-regulated in DCIS and IBC (Table 1) Red nodes represent the genes that were up-regulated in DCIS and IBC (Table 1), and empty nodes signify additional genes identified by IPA analysis due to their biological connection with the network based on evidence in the literature. (**A**) IPA network showing the genes whose expression is up-regulated in DCIS. The functional categorization for this network revealed Cell-To-Cell Signalling and Interaction and tissue development pathways. (**B**) IPA network representing genes up-regulated in IBC. The functional categorization of these genes revealed Cancer pathways, and the top biological function is cellular movement and cellular growth and proliferation. The full gene names for the gene symbols are listed in Table 1.

Studies have contributed to elucidating the molecular basis of DCIS progression, and several genes that are putatively involved in the development of an invasive phenotype in epithelial cells have been uncovered. To highlight the most promising candidate genes supposedly involved in DCIS progression, we gathered genes found in at least two independent epithelial cell-based studies [10,13,14,16,40,42–44,47–50] (Table 1). We concurrently used the core analysis tool of the IPA (Ingenuity Pathway Analysis software; Ingenuity Systems, Inc.) to increase the understanding of the regulatory interconnection among these genes and the most relevant biological processes. We evaluated the six up-regulated genes in DCIS and the 16 up-regulated genes in IBC cells (Table 1). The two most relevant networks were created (Figure 2). The first network connected the five genes that were up-regulated in DCIS with nine additional genes showing an enrichment of genes belonging to the Cell-To-Cell Signalling and Interaction pathway (Figure 2A). The central gene of this network is E-cadherin (CDH1), a highly characterized molecule. *CDH1*, which is expressed in DCIS epithelial cells and normal breast tissue, is a cell–cell adhesion protein that fulfils a prominent role in epithelial differentiation. A partial or total loss of *CDH1* expression has been repeatedly shown to occur in the transition from DCIS to IBC [52,53] and also correlated with a loss of differentiation characteristics, acquisition of invasiveness, increased tumour grade, metastatic behaviour and poor prognoses [51]. The mechanism by which *CDH1* expression is lost is currently not well established. Epigenetic silencing via promoter hyper-methylation seems to be a crucial mechanism of the transcriptional repression in breast cancer [54,55]. The second network functionally connected 16 genes that were up-regulated in IBC with 12 additional genes that display an enrichment of genes belonging to the Cancer pathway (Figure 2B). The most relevant biological functions for these genes are cellular movement, growth and proliferation. Genes involved in extracellular matrix remodelling (proteinases, collagenases and cysteine proteases) are clearly up-regulated at the IBC stage, where *MMP2* (matrix metalloproteinase 2) occupied a central role in the functional network. MMPs can degrade both the extracellular matrix and basement membrane, which are physical barriers that play important roles in preventing the expansive growth and migration of cancer cells. DNA demethlylation has an important role in cancer by turning on expression of pro-metastatic genes, such as the MMP2 [56]. The overexpression of MMPs in IBC is widely accepted to be associated with cancer-cell invasion and metastasis [43]. Loss of E-cadherin and gain of MMPs are well recognized as key mediators of the epithelial–mesenchymal transition, a mechanism closely associated with cell invasion, reinforcing the importance of both networks in the progression of DCIS. However, the exact role of these networks and their interactions with the other genes deserve to be deeper investigated in the transition of DCIS to IBC.

**Table 1:** Differentially expressed genes identified in DCIS showing up-regulation and down-regulation in epithelial cells between DCIS and IBC Compilation of differentially expressed genes in epithelial cells from DCIS and IBC selected from two independent studies. **DCIS**, ductal carcinoma *in situ*; **IBC**, invasive breast carcinoma, **IC**, *in situ* component

Gene Symbol	Description	Pure DCIS	IC-DCIS/IBC	IBC	References
*ADFP*	Adipose differentiation-related protein	UP	DOWN	DOWN	[10,47]
*ANAPC13*	Anaphase promoting complex subunit 13	UP	DOWN	DOWN	[10,47]
*ARHGAP19*	Rho GTPase activating protein 19	UP	DOWN	DOWN	[10,47]
*CLTCL1*	Clathrin, heavy chain-like 1	UP	DOWN	DOWN	[10,47]
*ANXA1*	Annexin A1	DOWN		UP	[42,48]
*CLDN1*	Claudin 1	DOWN		UP	[42,48]
*LUM*	Lumican	DOWN		UP	[43,49]
*MFAP2*	Microfibrillar-associated protein 2	DOWN		UP	[42,43]
*MMP2*	Matrix metalloproteinase-2	DOWN		UP	[42,43]
*SPARC*	Secreted protein, acidic, cysteine-rich (osteonectin)	DOWN		UP	[16,43]
*SARCL1*	SPARK-like 1(mast9, hevin)	DOWN		UP	[16,43]
*VIM*	Vimentin	DOWN		UP	[42,43,48]
*PLAU*	Plasminogen activator, urokinase	DOWN		UP	[13,14,42]
*BGN*	Biglycan	DOWN		UP	[13,42]
*FAP*	Fibroblast activation protein, alpha	DOWN		UP	[13,42]
*SFRP1*	Secreted frizzled-related protein 1	DOWN		UP	[14,50]
*RRM2*	Ribinucleotide reductase M2	DOWN		UP	[44,79]
*MMP11*	Matrix metalloproteinase-11	DOWN		UP	[13,43,44]
*COL1A2*	Collagen type I, alpha 2	DOWN		UP	[40,43]
*FBN1*	Fibrillin 1	DOWN		UP	[40,43]
*CDH1*	Cadherin 1, type 1	UP		DOWN	[42,48]
*GPCR11*	Coagulation factor II (thrombin) Receptor-like 1	UP		DOWN	[42,48]

In terms of genomic abnormalities, genes are recurrently amplified at certain chromosomal locations in the vast majority of breast cancers, indicating the common activation of some oncogenes during tumour development. DCIS displays genomic heterogeneity in the distinct immunophenotypes, similarly to genetic heterogeneity found in IBC [57]. Likewise, the majority of synchronous DCIS and IBC exhibit similar genomic sCNA profiles. However, additional genomic sCNAs occurred in the IBC component for a minority of the pairwise cases, suggesting a progression from DCIS to IBC [58].

Mutations in the *TP53* and *PIK3CA* genes have also been identified in DCIS. Mutations in *TP53* have been shown to occur more frequently in HG-DCIS compared with the LG subtype. These mutations are also more frequent in HER2-positive tumours than in ER/PR-positive tumours and TN DCIS [57]. However, *TP53* mutation has not been associated with the risk of DCIS progression. Mutations in the *PIK3CA* gene have been detected in both *in situ* and invasive matched breast samples [58]; however, the lower frequency or absence of *PIK3CA* mutation detected in the invasive component of some matched DCIS and IBC samples suggested that *PIK3CA* mutation is most likely an early event in breast tumorigenesis and is unlikely to play a role in DCIS progression [58].

Further studies of the genomic landscape of DCIS and IBC by aCGH (comparative genomic hybridization) and massively parallel sequencing are imperative for clarifying the genomic and genetic alterations involved in DCIS progression and discriminating the more aggressive phenotypes.

The role of miRNA (microRNAs) in cancer has been increasingly recognized. miRNAs exert their function by directly targeting downstream genes [59] and can function as either tumour suppressors [60] or oncogenes [61]. Thus, tumour formation, progression and metastasis may arise from a suppression of tumour suppressor miRNAs and/or overexpression of an oncogenic miRNA [62]. Although several studies have investigated miRNA in different aspects of breast cancer, such as in identifying differences in microRNA regulation between normal and tumour samples [63] in different tumour subtypes [64] and also in identifying prognostic biomarkers [65,66], few studies have investigated their role in the transition from DCIS to IBC. It has recently been discovered that some miRNAs are down- or overregulated in DCIS in comparison with normal histological breast tissue [67,68]. The miR-132 [67] has been observed to be underexpressed in DCIS, and in cell line assays, overexpression of miR-132 leads to inhibition of cell proliferation [68]. Interestingly, overexpression of miR-182 and miR-183, both overexpressed in DCIS in comparison with the normal, increased the expression of CBX7 (chromobox homologue 7), which in turn, positively regulate the expression of E-cadherin [68]. Thus, E-cadherin down-regulation towards the progression of DCIS to invasive disease might be result of combination of both promoter hypermethylation and action of miRNA. These findings point out to the importance for searching miRNAs as candidates to predict risk of DCIS progression.

## MICROENVIRONMENTAL CHANGES IN DCIS

The progression of DCIS to IBC is not only determined by molecular and genetic changes in epithelial cells but also strongly depends on microenvironmental factors [69]. Emerging evidence indicates that alterations, especially in myoepithelial [MECs (myoepithelial cells)] and stromal cells (fibroblasts and myofibroblasts), play a crucial role in the mechanism of the transition from DCIS to IBC, even at its earliest, pre-invasive stages.

Several recent studies have demonstrated that the progression of tumour epithelial cells to invade adjacent tissues can be promoted by fibroblasts and inhibited by myoepithelial cells. In this process, myoepithelial cells suppress tumour growth, whereas fibroblasts stimulate tumour growth [70,71].

Fibroblasts are one of the most crucial components of the tumour microenvironment, where they normally stimulate cell growth (through the production of growth factors and ECM proteins) and modulate immune polarization [70]. In this regard, fibroblasts are not only key players in the maintenance of normal tissue structure but also important in the progression and invasiveness of cancers. CAFs (carcinoma-associated fibroblasts) and normal fibroblasts have shown the differences in gene expression, mainly in genes involved in paracrine signalling, transcriptional regulation, the extracellular matrix, cell–cell interaction and cell adhesion/migration [72]. In addition, interactions between fibroblasts and epithelial cells seem to be reciprocal and lead to alterations in the gene expression profile of both cell types [73]. Furthermore, secretion of CXCL12 by CAFs may promote angiogenesis and increase cancer cell proliferation through interactions with CXCR4 expressed by tumour cells [74]. CAFs can promote the tumorigenic conversion of epithelial cells, whereas fibroblasts derived from normal tissue suppress this transition [75], reinforcing the existence of molecular modification of fibroblasts induced by epithelial tumour cells.

With respect to the differences in the fibroblasts that surrounds pure DCIS and pure IBC, fewer differences in the gene expression profile were observed compared with epithelial cells from both compartments [40].

In addition to their role in expelling milk from the ducts during lactation, MECs are also involved in the organizational development of the mammary gland through their effect on luminal epithelial cell polarity, branching, and differentiation [76]. Moreover, a major function of MEC is the synthesis and maintenance of the BM. The degradation of the BM is seen as a milestone for malignancy and invasion, and its disruption appears to coincide with the disappearance of the MEC. The inhibitory effect of MEC on cancer growth, invasiveness and angiogenesis has been demonstrated via the expression of a number of tumour suppressor proteins (maspin), ECM structural proteins (fibronectin and collagen), proteinase inhibitors (tissue inhibitor of metalloproteinase-1, TIMP-1), and angiogenic inhibitors (thrombospondin-1) [77]. In addition, MEC isolated from normal tissue have a distinct gene expression pattern compared with MECs from DCIS. MECs decreased the expression of genes involved in normal cell function, including thrombospondin, laminin and the oxytocin receptor, and increased the expression of genes that drive proliferation, migration, invasion and angiogenesis, including *CXCL12* and *CXCL14* [78]. These cells are thought to progressively lose their tumour suppressor function and disappear during the transition from DCIS to the invasive phenotype. Efforts have been made to define the role of MECs during the invasion process. Some authors advocate that BM disruption and neoplastic cell invasion are the result of changes in MECs, mainly the down-regulation of tumour suppressor genes. Another hypothesis states that as neoplastic luminal cells proliferate and the duct enlarges, the MEC population becomes insufficient for sustaining BM turnover, and luminal neoplastic cells are passively placed in the stroma with minimal changes in gene expression.

Of great interest is to uncover, in the transition of DCIS to IBC, the influence of the microenvironmental cells *per se* and also their influence on gene expression modulation in epithelial cells. In this sense, with the improvement in capturing and detecting molecular differences of single cells, progress in understanding this mechanism will be strengthened.

Thus, additional studies are crucial to define the role of microenvironment (myoepithelial and fibroblast-associated cells) in combination with epithelial cells in promoting the progression of DCIS to invasive disease.

## CONCLUSION AND PERSPECTIVES

Much progress has been made in characterizing the different types of DCIS at the molecular level, as well as the transition to invasive disease at the molecular and genetic level in the tumour epithelial cells. However, given the complexity of the mechanisms of progression and the evidence supporting the idea that this progression depends on the well-orchestrated action of the tumour epithelial and microenvironmental cells, our understanding is far from complete.

One of the major challenges is to define the molecular and genetic alterations of the three cell types present in the same tumour tissue–epithelial, MEC and fibroblast–and to understand the complex interactions among cells that collectively promote invasion into surrounding mammary tissue. Continuous advances in the tools to assess molecular alterations in these cells are fundamental for the successful development of optimal treatments for DCIS.

Although independent groups and datasets have confirmed the involvement of molecular and genetic factors in the progression of DCIS, none can currently be considered robust enough to be used as molecular markers for risk stratification in patients with DCIS. Multidisciplinary teams must properly validate these findings before this knowledge can be transferred into clinical practice, where it could be used to predict the risk of DCIS progression. This practice would offer women with DCIS an individually tailored treatment that is minimally aggressive and has a maximal cure rates.
